# Virus-like attachment sites as structural landmarks of plants retrotransposons

**DOI:** 10.1186/s13100-016-0069-5

**Published:** 2016-07-28

**Authors:** Edgar Andres Ochoa Cruz, Guilherme Marcello Queiroga Cruz, Andréia Prata Vieira, Marie-Anne Van Sluys

**Affiliations:** Departamento de Botânica, Instituto de Biociências (IB), Universidade de São Paulo (USP), 05508-090 São Paulo, SP Brasil

**Keywords:** LTR-RTs, Angiosperm genomes, *vl-att* site, Retrotransposons

## Abstract

**Background:**

The genomic data available nowadays has enabled the study of repetitive sequences and their relationship to viruses. Among them, long terminal repeat retrotransposons (LTR-RTs) are the largest component of most plant genomes, the Gypsy and Copia superfamilies being the most common. Recently it has been found that Del lineage, an LTR-RT of Gypsy superfamily, has putative virus-like attachment (*vl-att*) sites. This signature, originally described for retroviruses, is recognized by retroviral integrase conferring specificity to the integration process.

**Results:**

Here we retrieved 26,092 putative complete LTR-RTs from 10 lineages found in 10 fully sequenced angiosperm genomes and found putative *vl-att* sites that are a conserved structural landmark across these genomes. Furthermore, we reveal that each plant genome has a distinguishable LTR-RT lineage amplification pattern that could be related to the *vl-att* sites diversity. We used these patterns to generate a specific quick-response (QR) code for each genome that could be used as a barcode of identification of plants in the future.

**Conclusions:**

The universal distribution of *vl-att* sites represents a new structural feature common to plant LTR-RTs and retroviruses. This is an important finding that expands the information about the structural similarity between LTR-RT and retroviruses. We speculate that the sequence diversity of *vl-att* sites could be important for the life cycle of retrotransposons, as it was shown for retroviruses. All the structural *vl-att* site signatures are strong candidates for further functional studies. Moreover, this is the first identification of specific LTR-RT content and their amplification patterns in a large dataset of LTR-RT lineages and angiosperm genomes. These distribution patterns could be used in the future with biotechnological identification purposes.

**Electronic supplementary material:**

The online version of this article (doi:10.1186/s13100-016-0069-5) contains supplementary material, which is available to authorized users.

## Background

Since the genome of *Arabidopsis thaliana* was sequenced in 2000, 55 other plant genomes have been released and published [1, 2]. This has advanced our understanding of genome composition, such as the discovery that repetitive sequences are major constituents of most genomes [3]. Among these repetitive sequences are the transposable elements (TEs), which are mobile genetic sequences present in plants and in all eukaryotes. TEs comprise approximately 45 % of the human genome and form the vast majority of the total DNA content of most plant genomes, in some cases reaching close to 80 % [4–6].

The predominant TE found in plant genomes is the long terminal repeat retrotransposons (LTR-RTs). For example, it represents ~79 % of the maize (~2.3 Gb total) and ~55 % of the sorghum (~730 Mb total) genomes [7–11]. Based on sequence similarities and on the structural/domains organization, LTR-RTs are divided into two major superfamilies: the Gypsy and the Copia [3]. Phylogenetic analysis of the reverse transcriptase domain revealed that the Gypsy superfamily is divided into five lineages, namely Athila, CRM, Del, Galadriel, and Reina, while the Copia superfamily is divided into six lineages (Ale, Angela, Bianca, Ivana, Maximus, and Tar) [12–14]. It has been shown by coding sequence and structural similarities that LTR-RTs are related to retroviruses [15], it has been suggested that retroviruses evolved from the Gypsy superfamily after acquisition of the envelope gene [16].

Our research on the relationship between retroviruses and LTR-RTs has recently revealed that Del has putative virus-like attachment (*vl-att*) sites in its LTRs [17–19]. The LTRs are direct repeat sequences located at the 5′ and 3′ ends of the LTR-RT elements containing the regulatory information of the LTR-RT such as promoters, enhancers and termination signals [20]. The *att* sites were originally described in retroviruses as sequences recognized by retroviral integrase to confer specificity to the integration process [17, 18, 21]. We questioned whether *vl-att* sites are specific to the Del lineage or are conserved structural landmarks across plant LTR-RTs and, therefore, a new structural feature common to plant LTR-RTs and retroviruses. To study this hypothesis, we retrieved all the putative complete elements, a total of 26,092 elements, from the other LTR-RTs lineages present in the 10 angiosperm genomes used previously to study the Del lineage [19].

The present study supports the existence of structural *vl-att* sites in nine out of 10 LTR-RT lineages of 10 angiosperm genomes. We also propose a multivariable genome-specific LTR-RTs “barcode” signature for the putative complete LTR-RTs content and their differential amplification pattern to identify each genome analyzed. The differential amplification patterns found could be related to the *vl-att* sites diversity we discovered. To our knowledge such a wide landscape of LTR-RT and angiosperm genomes was never considered to reveal, simultaneously, the existence of structural *vl-att* site signatures and the genome-LTR-lineage amplification patterns that we describe herein.

## Results and discussion

### Establishing a conserved structural retrovirus landmark on plant retrotransposons: the virus-like attachment sites (*vl-att*)

In order to have a representative sample of the angiosperm genomes, we used the five eudicot (*Arabidopsis thaliana, Medicago truncatula, Populus trichocarpa, Vitis vinifera and Glycine max*) and the five monocot species (*Brachypodium dystachyon, Oryza sativa*, *Setaria italica, Sorghum bicolor,* and *Zea mays*) examined previously by our group [19]. They were analyzed with the LTR_STRUC software [22], which finds full length LTR-RT elements based on structural and sequence criteria. We identified 28,622 putative complete elements (Table 1), defined as those presenting two intact LTRs. LTR_STRUC software, which is only effective for full-length LTR retrotransposons [22], generated the primary data composed of 28,622 LTR-RT elements where the *vl-att* sites were analyzed.

**Table 1:** Total copy-number of putative complete LTR-retrotransposons identified in each genome and classified according to lineage

Plant genomes		Putative complete elements copy-number by lineage											Total copy number per genome	Genome size database (MB)	% GC content per genome
		Ale	Angela	Bianca	Ivana	Maximus	Tar	Athila	CRM	Del	Galadriel	Reina			
Eudicot	At	12	2^a^	5^a^	15	7^a^	12	24	3^a^	11	0	9	83	119	36
Eudicot	Mt	36	8^a^	4^a^	39	103	49	148	4^a^	57	0	14	446	291	36
Eudicot	Pt	125	2^a^	1^a^	70	0	29	31	23	3^a^	6	41	325	378	34
Eudicot	Vv	743	106	75	78	29	173	368	31	12	36	47	1698	486	35
Eudicot	Gm	87	199	0	276	862	168	951	767	72	0	390	3772	950	35
Monocot	Bd	69	61	14	47	11	7^a^	191	25	12	0	30	460	271	46
Monocot	Os	88	55	2^a^	68	50	121	642	31	262	1	84	1402	372	44
Monocot	Si	112	457	0	42	10	40	605	122	191	0	43	1622	392	46
Monocot	Sb	186	65	31	172	350	19	2984	192	621	0	208	4828	659	44
Monocot	Zm	294	197	24	138	5102	73	6105	320	1289	0	396	13,938	2066	47
Total copy number per lineage		1752	1152	156	945	6524	691	12,049	1518	2530	43	1262	28,622		
Superfamily		Copia	Copia	Copia	Copia	Copia	Copia	Gypsy	Gypsy	Gypsy	Gypsy	Gypsy			

This table indicates the putative complete LTR-RT elements copy-number identified in each genome (including the already described Del lineage). It also shows the size and GC content of the ten fully sequenced genomes used (*A. thaliana -* At*, M. truncatula -* Mt*, P. trichocarpa -* Pt*, V. vinifera –* Vv, *G. max -* Gm*, B. distachyon -* Bd*, O. sativa –* Os, *S. italica -* Si*, S. bicolor –* Sb and *Z. mays -* Zm*,*). ^a^ Represents the elements from a particular lineage in a genome that could not be used for the *vl-att* sites analyses, because of the low copy-number (≤8 copies)

**Fig. 1:**
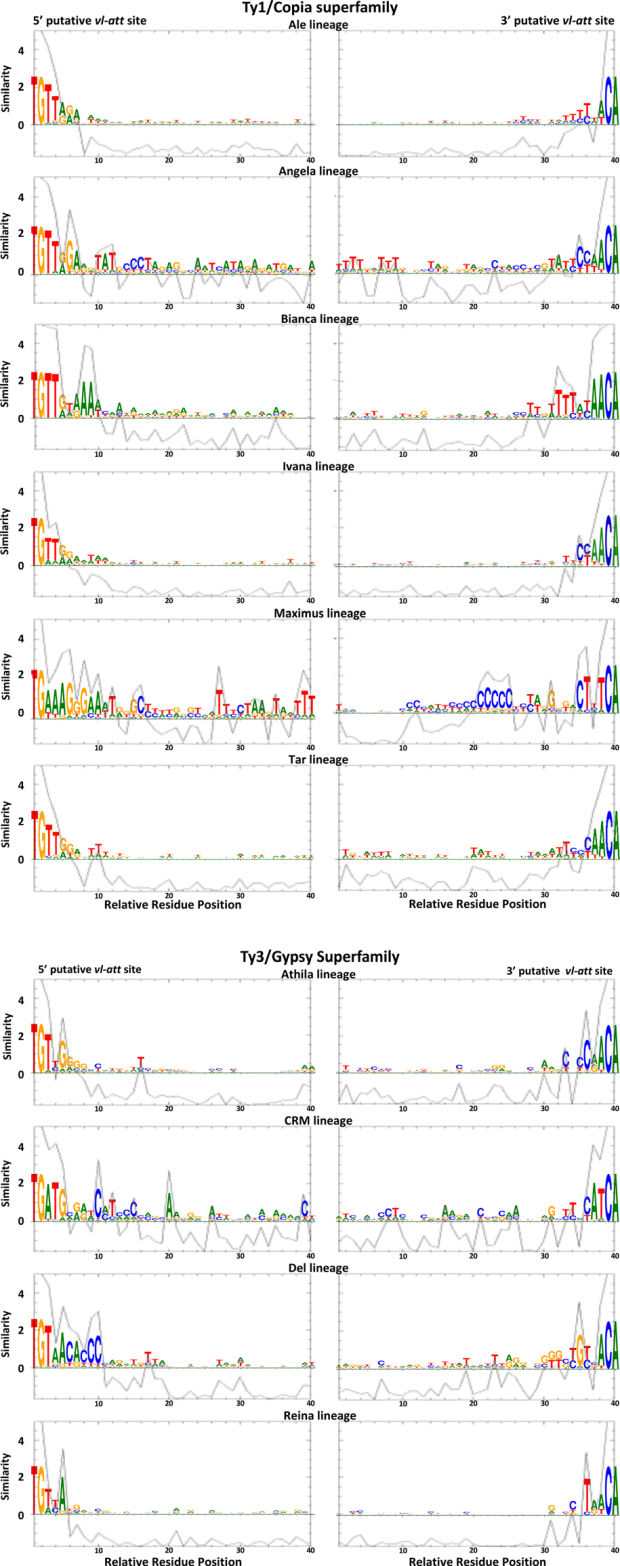
Sequence logos and PlotCon of U3 and U5 *vl-att* putative sites of 9 LTR-retrotransposon lineages. Sequence logos of the first and last 40 bases of the LTR from 9 LTR-RT lineages found in ten fully sequenced genomes (*A. thaliana -* At*, M. truncatula -* Mt*, P. trichocarpa -* Pt*, V. vinifera –* Vv, *G. max -* Gm*, B. distachyon -* Bd*, O. sativa –* Os, *S. italica -* Si*, S. bicolor –* Sb and *Z. mays -* Zm*,*). Sequence logo is a graphical representation of nucleic acid multiple sequence alignment. Each logo consists of stacks of symbols, one stack for each position in the sequence. The overall height of the stack indicates the sequence conservation at that position, while the height of symbols within the stack indicates the relative frequency of each nucleic acid at that position. Behind each logo it is the PlotCon analysis, where the X-axis for all plots refers to the relative residue position in each alignment and the Y-axis to their similarity, indicated as the pairwise scores that are taken from the specified similarity matrix. The PlotCon graphics are based on an algorithm that shows, along the alignment, the regions with significant similarity (above 0 mark of similarity), giving a strong view of the *vl-att* sites candidates

Next, we isolated the 5′ and 3′ ends of the 26,092 elements from the LTR-RTs lineages detected in the studied angiosperm genomes and of the 2530 elements from the Del lineage as a control. The LTR region is structurally composed of three regions, namely the U3, R, and U5 regions. The promoter and other regulatory sequences are located within U3 [23]. The *vl-att* should be at the beginning of the 5′ U3 region and at the end of the 3′ U5 region [19]. Using WebLogo and PlotCon [24, 25] to analyze the 40 initial and terminal bases from the LTRs we identified conserved regions for most of the lineages (Fig. 1). The results given by PlotCon are based on an algorithm that shows, along the alignment, regions with significant similarity (above 0 indicates similarity) and can therefore detect putative *vl-att* sites, which are good candidates for further functional studies [17, 18, 21, 26]. Considering the results of the two analyses and the number of sequences used, it is clear that conserved regions compatible with *vl-att* sites are structurally present in the LTR of each lineage studied. Only the Galadriel lineage did not show regions clearly compatible with *vl-att* sites, most probably because plant genomes have a low copy number of this lineage (43 copies total from only three genomes).

Figure 1 displays the conserved regions and the similarities identified along the putative *vl-att* sites. Four of the studied lineages presented a clear segment of high similarity that established the length of the structural *vl-att* sites hereby described: Ale (7 bp-6 bp), Bianca (13 bp-13 bp), Ivana (5 bp-6 bp) and Reina (5 bp-7 bp). The Tar and Athila lineages exhibited a conserved nucleotide stretch of five bases and an additional conserved nucleotide outside this region. Our results are compatible with the length reported for the structural *vl-att* sites from the Del lineage (10 bp-11 bp) [19]. Long segments presenting high similarity levels were detected in Angela (18 bp-10 bp), Maximus (16 bp-5 bp), and CRM (12 bp-10 bp), making it more difficult to establish the correct length of the structural *vl-att* sites of these lineages. The criterion used to delimit these long structural *vl-att* sites is the presence of a maximum of two gaps, not longer than two nucleotides, in the high-similarity region.

The structural *vl-att* sites are conserved across all the angiosperm genomes and across all the 10 retrotransposons lineages analyzed (Fig. 1 and Additional file 1: Figure S1). Ale, Bianca, Ivana and Reina structural *vl-att* sites are highly conserved across the analyzed genomes with only minor nucleotide and size differences (ranging from 1pb to 3 bp), except for the *Zea mays* genome (Fig. 1 and Additional file 1: Figure S1). In the *Zea mays* genome, Bianca and Ivana lineages display putative *vl-att* sites with a longer similarity region (40 bp) than the average length described herein for the other lineages (Additional file 1: Figure S1). Twenty-four copies in Bianca and 138 copies in Ivana lineages support these structural *vl-att* sites (Table 1).

The Athila and Tar lineages presented less homogeneous lengths (differences greater than 3 bp) between their structural *vl-att* sites general signature (Fig. 1) and the specific structural *vl-att* sites of some specific genomes and plant groups (Additional file 1: Figure S1). Finally, although the elements with long high-similarity regions (detected in the Angela, Maximus and CRM lineages) varied in length among the genomes and plant groups, most of the nucleotides included in these regions were conserved (Additional file 1: Figure S1). These are interesting results because they indicate that some structural *vl-att* sites are not only lineage specific but also lineage-genome specific. All the putative *vl-att* site signatures presented herein are strong candidates for further functional studies. Genome-specific analysis was not possible for genomes carrying a lineage with a low copy number of complete LTR-RT elements (≤8 copies; see Table 1 for details).

To our knowledge, this is the first report indicating that structural *vl-att* landmarks are not of Del lineage particularity since nine out of 10 LTR-RT lineages studied also display them. The Galadriel lineage was not considered in our study due to its low copy number (43 copies) and restricted distribution. The number of putative complete elements used varied from 156 to 12,049 per lineage (Table 1). The sample validation of these genomes, which will be discussed in the next section, and the significant similarity of the alignments showed by the PlotCon analyses support the notion of structural *vl-att* sites landmarks. Six structural *vl-att* sites are clearly short as was the already described Del structural *vl-att* sites, while other three could have extended length. Because the structural *vl-att* sites described herein are specific in length and nucleotide composition for each lineage, it is possible that they have a role in retrotransposon speciation and life cycle. Moreover, they may be responsible for the differential amplification pattern of these lineages in the studied genomes, as the ones that will be shown in the next section of this work.

Our study highlights the presence of putative *vl-att* sites along LTR-RTs in plants, these are specific to each lineage and in some cases also to each genome, and warrants further research on the importance of the *vl-att* sites for each lineage integrase recognition specificity in the LTR-RTs replication cycle. Indeed, the specificity to the integration process conferred by the recognition of *att* sites by the retroviral integrase is reported for retroviruses [18, 21] and should be clarified in retrotransposons. Moreover, it would be interesting to investigate the presence of *vl-att* sites in genomes other than plants.

### Exploring LTR-RT amplification patterns that might be linked to the diversity of structural virus-like attachment sites (*vl-att*)

We postulated that lineage-specific *vl-att* site signatures could have functional implications for the amplification of LTR-RT elements. For instance, *att* sequences of retroviruses are recognized by the retroviral integrase to confer specificity to the integration process [17, 18, 21]. To test this hypothesis, we analyzed the amplification pattern of the 28,622 putative complete LTR-RT elements used in the *vl-att* site analyses. These elements were categorized as matching one of the six Copia or one of the four Gypsy lineages (Table 1). This classification was performed using hmmer alignment against previously described Hidden Markov Model (HMM) profiles, which were created using alignments of lineages reverse transcriptase amino acids [12]. Table 1 also includes the 2530 elements of the Del lineage (Gypsy) used herein for comparative purposes [19].

**Fig. 2:**
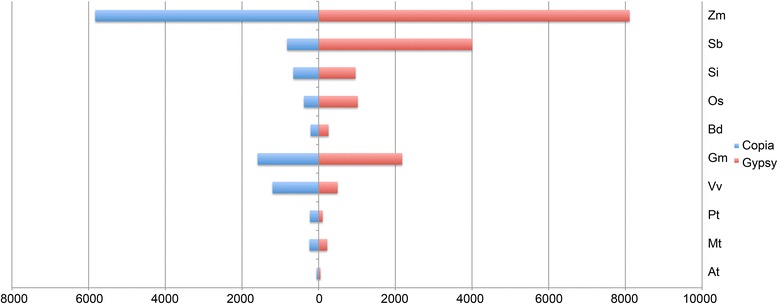
Histogram representing the copy-number of putative complete LTR-retrotransposons divided by superfamilies, which were found in 10 plant genomes. The ten fully sequenced genomes used (*A. thaliana -* At*, M. truncatula -* Mt*, P. trichocarpa -* Pt*, V. vinifera –* Vv, *G. max -* Gm*, B. distachyon -* Bd*, O. sativa –* Os, *S. italica -* Si*, S. bicolor –* Sb and *Z. mays -* Zm*,*) are shown divided by its LTR-RT superfamilies content, Copia (blue) and Gypsy (red)

The *Zea mays* genome has the highest number of elements because Athila (Gypsy) has 6105 copies, followed by Maximus (Copia) with 5102 copies and finally Del (Gypsy) with 1289 copies (Table 1). *Sorghum bicolor* comes after *Zea mays* in terms of LTR-RT amplification. Indeed, Athila is highly represented in sorghum, albeit with approximately half of the copies found in *Zea mays*, followed by Del and Maximus (Table 1). Another genome with a high copy-number of elements is the eudicot plant *Glycine max* (Fig. 2 and Table 1). Interestingly, the studied monocots have almost four times more putative complete LTR-RT elements than the studied eudicot species (22,259 and 6363 LTR-RTs, respectively). Furthermore, the Gypsy superfamily is 1.5 times more represented in the studied genomes than the Copia superfamily (17,402 and 11,220 LTR-RTs, respectively). Taken together, these results reveal two interesting trends worthy of notice: (i) as the genome size increases the number of LTR-RTs also increases, which confirm previous findings [27–30]; and (ii) grasses carry more putative complete LTR-RTs than the other studied genomes (Fig. 2 and Table 1).

Bianca (Copia) and Galadriel (Gypsy) lineages are poorly represented in the analyzed genomes, totaling 199 copies. The monocot *Brachypodium dystachyon* and the eudicot *Arabidopsis thaliana* are the genomes with the lowest copy-numbers of putative complete LTR-RT elements (Fig. 2 and Table 1).

The more frequent occurrence of high copy-numbers of LTR-RTs found in some grasses genomes (e.g., *Zea mays* and *Sorghum bicolor*) and the presence of low copy-numbers observed in monocot and eudicot plant groups (e.g., *Brachypodium dystachyon* and *Arabidopsis thaliana*) are in accordance with previous studies employing complete and non-complete LTR-RTs elements. These previous studies only used some of the genomes or lineages analyzed herein [8, 9, 13, 31]. Furthermore, the copy-number reported here for the Copia superfamily (ordered from the most to the least frequently represented lineages: Athila, Maximus, Del, Ale) corroborates with recent studies [12, 32], one of which used fluorescent *in situ* hybridization to analyze lineages from both Copia and Gypsy superfamilies using complete and non-complete LTR-RT elements [12]. Therefore, we believe that the LTR-RTs sampling performed here with the LTR_STRUC software was effective and has allowed us to expand the current understanding about the amplification of the LTR-RT lineages among the genomes studied, regardless of the software structural analyses that enriches the sampling with recent events of amplification.

**Fig. 3:**
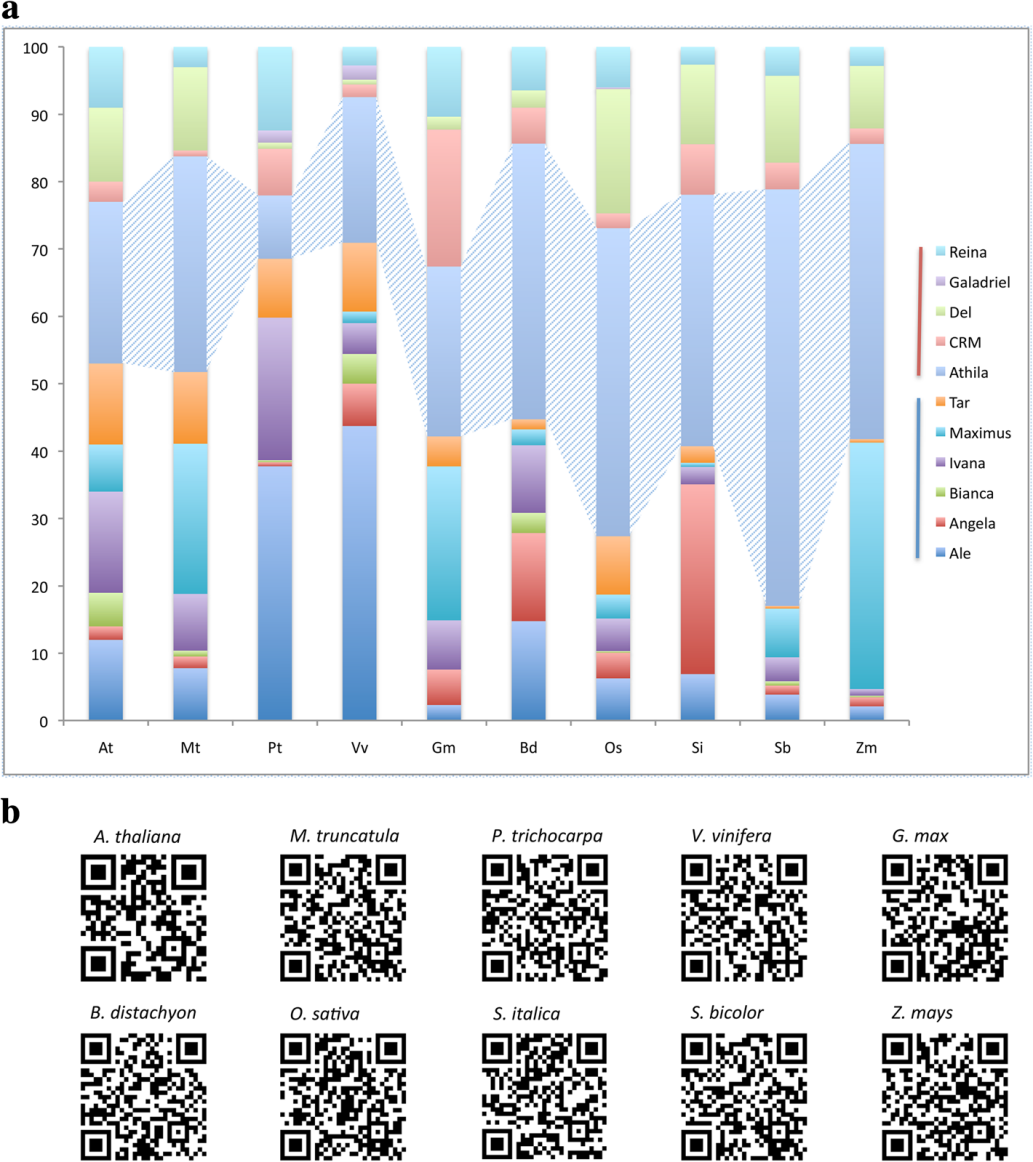
Normalized copy number of putative complete LTR-retrotransposons divided in 11 LTR-retrotransposon lineages, which were found in 10 plant genomes. **a** Histogram representation – The Copia (blue line lineages) and Gypsy (red line lineages) are shown. Each LTR-RT lineage is represented by different colors along the histogram of the LTR content from ten fully sequenced genomes (*A. thaliana -* At*, M. truncatula -* Mt*, P. trichocarpa -* Pt*, V. vinifera –* Vv, *G. max -* Gm*, B. distachyon -* Bd*, O. sativa –* Os, *S. italica -* Si*, S. bicolor –* Sb and *Z. mays -* Zm*,*). **b** QR-code representation – For each genome a QR-code was generated using the normalized data (Fig. 3a and Table 2), which represents each lineage contribution to each specific studied genome. The code can be read using a common cell-phone QR-code scanner

The “total copy-number” data presented on Fig. 2 and Table 1 was normalized to compare the contribution of each lineage to the content of LTR-RTs across genomes ((lineage copy-number in a genome X 100)/ copy-number of all the putative complete LTR-RTs in the same genome). As shown in Fig. 3a and Table 2, the order of copy-number from the most to the least frequently represented lineages (Athila, Maximus, Del, Ale) was not maintained once the data was normalized (Athila, Ale, Maximus, Angela, Ivana, CRM, Del). Also, the impact of the Athila lineage on the content of LTR-RTs in the genome of *Sorghum bicolor* was stronger than in *Zea mays*, representing 61.8 % versus 43.8 % of the LTR-RTs content, respectively (Fig. 3a and Table 2). This is particularly interesting given that Athila’s “total copy-number” in *Sorghum bicolor* is lower than in *Zea mays* (Table 1). This shows that the normalization of the data is a fundamental step because the contributions of lineage and genomic LTR-RTs are not obvious or could be misunderstood when only the “total copy-number” of an individual lineage/genome is considered.

**Table 2:** Normalized number of putative complete LTR-retrotransposons identified in each genome and classified by lineage

Plant genomes	Putative complete elements genome contribution by lineage (%)										
	Ale	Angela	Bianca	Ivana	Maximus	Tar	Athila	CRM	Del	Galadriel	Reina
At	12.0	2.0	5.0	15.0	7.0	12.0	24.0	3.0	11.0	0.0	9.0
Mt	7.8	1.7	0.9	8.4	22.3	10.6	32.0	0.9	12.3	0.0	3.0
Pt	37.8	0.6	0.3	21.1	0.0	8.8	9.4	6.9	0.9	1.8	12.4
Vv	43.8	6.2	4.4	4.6	1.7	10.2	21.7	1.8	0.7	2.1	2.8
Gm	2.3	5.3	0.0	7.3	22.9	4.5	25.2	20.3	1.9	0.0	10.3
Bd	14.8	13.1	3.0	10.1	2.4	1.5	40.9	5.4	2.6	0.0	6.4
Os	6.3	3.9	0.1	4.8	3.6	8.6	45.7	2.2	18.7	0.1	6.0
Si	6.9	28.2	0.0	2.6	0.6	2.5	37.3	7.5	11.8	0.0	2.7
Sb	3.9	1.3	0.6	3.6	7.2	0.4	61.8	4.0	12.9	0.0	4.3
Zm	2.1	1.4	0.2	1.0	36.6	0.5	43.8	2.3	9.2	0.0	2.8
Superfamily	Copia	Copia	Copia	Copia	Copia	Copia	Gypsy	Gypsy	Gypsy	Gypsy	Gypsy

This table indicates the normalized copy-number, as percentages, of LTR-RT elements identified in each genome (including the already described Del lineage) from the ten fully sequenced genomes used (*A. thaliana -* At*, M. truncatula -* Mt*, P. trichocarpa -* Pt*, V. vinifera –* Vv, *G. max -* Gm*, B. distachyon -* Bd*, O. sativa –* Os, *S. italica -* Si*, S. bicolor –* Sb and *Z. mays -* Zm*,*). The normalization used the LTR-RT total copy-number in each genome as 100 %

In other cases, the normalized and non-normalized data (Table 2 and 1, respectively) were coincident, as for the three Copia superfamily lineages that showed to be important size contributors to some of the genomes (Ale 43 % - *Vitis vinifera*, Angela 28.2 % - *Setaria italica* and Maximus 36 % - *Zea mays*). While in the three Gypsy lineages that proved to be important size contributors (Athila, CRM and Del), only CRM in *Glycine max* showed the same profile after normalization. Thus, the lineage genome-contribution signature for these four cases is maintained not only as “total copy-number” but also as a lineage contribution to the LTR-RTs genome content (Tables 1 and 2).

Furthermore, the Gypsy superfamily is more represented in the studied plant genomes than the Copia superfamily, both in terms of “total copy-number” and as the major contributor to the LTR-RTs content (normalized data not shown). This is confirmed by previous studies using complete and non-complete LTR-RTs elements and analyzing up to a maximum of three different plant genomes, but never in the complete angiosperm and lineages dataset explored herein [8, 11, 12, 14]. Once again, these data validates the sampling of LTR-RTs of the studied genomes using the LTR_STRUC software. The copy-number ratios of these superfamilies were also shown for the apple tree *Malus domestica* genome using dot blot hybridizations [33]. However, our normalized data showed that Copia lineages contribute most to the LTR-RTs content of the eudicot species, whereas the Gypsy lineages contribute most to the LTR-RTs content of the studied monocot species (Fig. 3a and Table 2).

LTR-RT elements are widely and abundantly present in plant genomes and have been implicated in their evolution [7–9, 30]. Here we present the LTR-RTs amplification as a function of the “total copy-number” and quantified the relative contribution of each lineage to the content of LTR-RTs of each genome through data normalization (Table 2 and Fig. 3a). We focused on putative complete LTR-RTs insertions and did not consider the copies affected by recombination and decay, which are common events on the elements’ life cycle. Nevertheless, our “total copy-number” ratios (Gypsy vs. Copia) matched the data presented in previous studies considering complete and incomplete LTR-RTs copies, which also represent different stages of the elements’ life cycle [27–30].

The data presented above suggest that the studied LTR-RTs lineages have a particular amplification pattern in each of the genomes, which may be linked to the diversity of the putative *vl-att* sites found. The normalized data simplified the comparison of the LTR-RTs amplification patterns, because it considered the contribution to the LTR-RTs content in each genome instead of the raw “total copy-number” (Fig. 3a and Table 2). It allowed us to propose a multivariable genome-specific LTR-RTs “barcode” signature, which gives an overview of the putative complete LTR-RTs content and their differential amplification in the studied genomes (Fig. 3a, b and Table 2). For instance, the barcode offered an easy way to identify the importance of the Ale lineage to the LTR-RTs content in *Populus trichocarpa* and *Vitis vinifera*, the latter being the only perennial species used in our study. It also indicated that Athila is an important component of the LTR-RTs content for most of the studied genomes (Fig. 3a). To our knowledge, this is the first comparative analysis of specific LTR-RT content and their amplification patterns in a large dataset of fully sequenced angiosperm genomes, allowing a deeper understanding of the relationship between these lineages and these genomes as never before.

Based on our normalized data we generated specific identification QR-code for each genome that can be revealed using a common cell-phone QR-code scanner (Fig. 3b). The effective contribution of the proposed LTR-RTs-barcode depends on the capacity to distinguish between plant species even more closely related. However, the closest species used in this study, in terms of evolutionary distances, are *Zea mays* and *Sorghum bicolor* (11.9 million years ago – Mya) [34]. The LTR-RTs-barcode differences between these species were readily detected herein. Further research will be needed to confirm the effectiveness of the proposed barcode system using genomes with smaller evolutionary distances. The likelihood is high because studies using closely related plant species have shown differential amplification of genomic LTR-RTs [27, 35–37]. Our LTR-RTs barcode system is based on data not explored before, the diversity of putative *vl-att* site signatures and the differential amplification pattern of 11 LTR-RT lineages in 10 fully-sequenced genomes. The QR-code proposed here illustrates how this concept could be used in the future as a biotechnological tool for identification of commercially valuable cultivars especially given that the cost of genome sequencing is reducing faster than expected by the Moore’s Law [38].

## Conclusion

Analysis of 26,092 putative complete elements representing 10 LTR-RT lineages of 10 different angiosperm genomes allowed us to find putative *vl-att* sites in nine out of 10 lineages. The present study is the first to show that *vl-att* sites are structural conserved landmarks in LTR-RTs across distantly related angiosperms. This is an important finding that expands the information about the structural similarity between LTR-RT and retroviruses. We speculate that the sequence diversity of *vl-att* sites may be important for the life cycle of retrotransposon and amplification patterns of these lineages in the genomes of angiosperms analyzed herein. Future functional studies of these sequences are necessary to test this hypothesis. Here we reveal three distinct patterns in the structural *vl-att sites*: (i) four lineages (Ale, Reina, Bianca and Ivana) have minor nucleotide differences among their sequence regardless of the angiosperm genome considered (ii) two lineages (Athila and Tar) display marked differences and (iii) three lineages (Angela, Maximus and CRM) with long structural *vl-att* varied widely in size but little in nucleotide sequence.

The current study also describes the amplification patterns of the 10 LTR-RTs lineages along these plant genomes using a methodology that allows novel observations such as the grasses genomes carry more putative complete LTR-RTs than the other studied genomes. Also, “total” vs “relative” abundance illustrates the singularity of LTR-RT amplification pattern in each genome. Finally, from our data a specific QR-code identification system was derived for each of the angiosperm genomes that can be used with a common cell-phone QR-code reader. The QR-code proposed may have biotechnological applications in the identification of commercially valuable cultivars.

## Methods

### Element extraction and classification

Ten fully sequenced genomes (*A. thaliana* - At - AtGDB171/TAIR9 – GenBank current version is TAIR10 at GCA_000001735.1*, M. truncatula* - Mt – Mt3.5 – GenBank current version is MedtrA17_4.0 at GCA_000219495.2*, P. trichocarpa* - Pt - Ptr v2.2 – GenBank current version is Poptr2_0 at GCA_000002775.2*, V. vinifera* - Vv - Genoscope 12X – same GenBank current version at GCA_000003745.2, *G. max* - Gm – Glyma1 – GenBank current version is Glycine_max_v2.0 at GCA_000004515.3*, B. distachyon -* Bd – Version1 – GenBank current version is Brachypodium_distachyon_v2.0 at GCA_000005505.2*, O. sativa* - Os – Release 7 – GenBank current version is Build 4.0 at GCA_000005425.2, *S. italica –* Si - JGI 8x v2 Sitalica_164 – GenBank current version is Setaria_italica_v2.0 at GCA_000263155.2*, S. bicolor* - Sb - JGI Sbi1 – GenBank current version is Sorbi1 GCA_000003195.1 and *Z. mays* - Zm - B73_RefGen_v2 – GenBank current version is B73 RefGen_v3 at GCA_000005005.5) were downloaded (11/25/2011) from the plandGDB ftp website [39]. The complete genome sequences were split into sequences from individual chromosomes and screened using LTR_STRUC [22] with default parameters. Hidden Markov Model (HMM) profiles were built using the HMMER package (version 2.3.2) based on reverse transcriptase amino acid alignments as previously described [12]. Extracted sequences were conceptually translated in all six frames and subjected to HMMscan (HMMER 2.3.2 package) against the HMM profiles, with an e-value cut-off at 1e^−10^. All sequences were classified into lineages [12] according to the best hit. Further analyses were performed only on complete putative elements, which were defined as elements with two intact LTRs found by the LTR_STRUC software. Using our normalized data results, we generated a specific QR identification code for each genome, using the Barcode generator online tool (http://www.barcode-generator.org/). A local database was built at GaTE lab (https://gate.ib.usp.br/GateWeb/) and sequences are available upon request.

### Identifying structural virus-like attachment (*vl-att*) sites

Two conserved regions were identified along most LTR-RT lineages by examining alignments of all sequences in Jalview (version 2.4.0.b2) using the option “color *per* conserved sites” [40]: one at the 5’ end of the LTR and a second at the 3′ end of the LTR,. The first and last 40 bases of the LTRs were submitted to WebLogo [24] and PlotCon, both of which are part of the EMBOSS Molecular Biology software analysis package (6.3.1) [25], to examine and plot the sequence conservation analysis results. The PlotCon algorithm represents the alignment quality quantification, helping to determine the relevant extension of each putative *vl-att* site. When the conservation exceeded 40 bp, 150pb was used. Nevertheless, alignment-quality gaps were found in the structural *vl-att* sites. To detect the strongest candidates, we selected structural *vl-att* sites with a maximum of two quality-gaps per sequence and a maximum of two nucleotides of quality-gap extension.

## Additional file

Additional file 1: Figure S1. Sequence logos and PlotCon of U3 and U5 *vl-att* putative sites of 9 LTR-retrotransposon lineages divided by genome and plant group. Sequence logos of the first and last 40 bases of the LTR from 9 LTR-RT lineages divided by genome or plant group (eudicot - monocot species). Sequence logo is a graphical representation of nucleic acid multiple sequence alignment. Each logo consists of stacks of symbols, one stack for each position in the sequence. The overall height of the stack indicates the sequence conservation at that position, while the height of symbols within the stack indicates the relative frequency of each nucleic acid at that position. Behind each logo it is the PlotCon analysis, where the X-axis for all plots refers to the relative residue position in each alignment and the Y-axis to their similarity, indicated as the pairwise scores that are taken from the specified similarity matrix. The PlotCon graphics are based on an algorithm that shows, along the alignment, the regions with significant similarity (above 0 mark of similarity), giving a strong view of the *vl-att* sites candidates. (PDF 8527 kb)

## Abbreviations

HMM: Hidden markov model
LTR-RT: LTR retrotransposon
QR: Quick response
vl-att: Virus like attachment site
