# Supplementary material for: Virus-like attachment sites as structural landmarks of plants retrotransposons
Source: Mob DNA. 2016 Jul 28;7:14. doi: 10.1186/s13100-016-0069-5 (PMC4963935; doi:10.1186/s13100-016-0069-5)

Fig. S1

Ale lineage *vl-att* sites in Eudicots

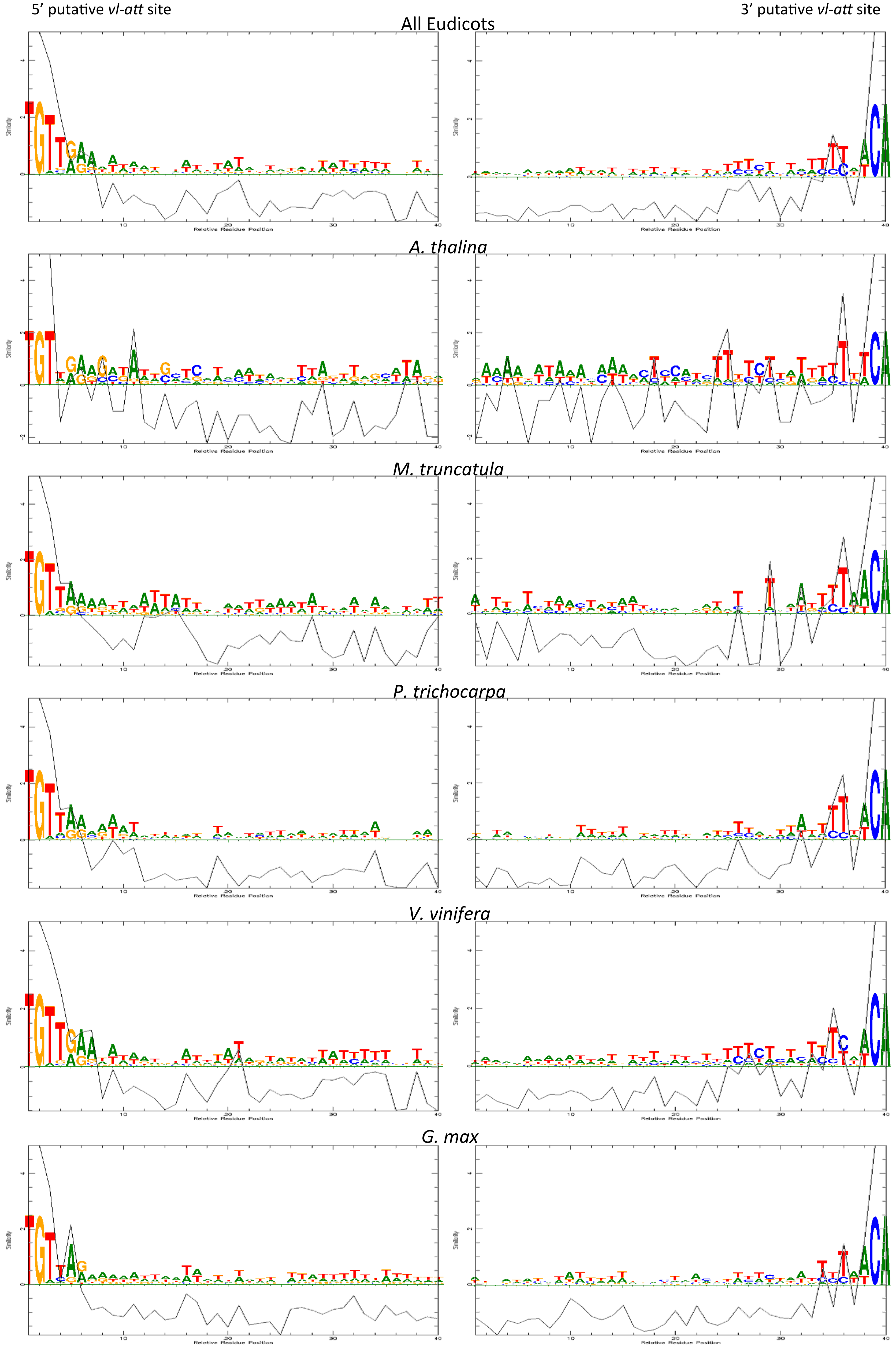

Ale lineage *vl-att* sites in Monocots

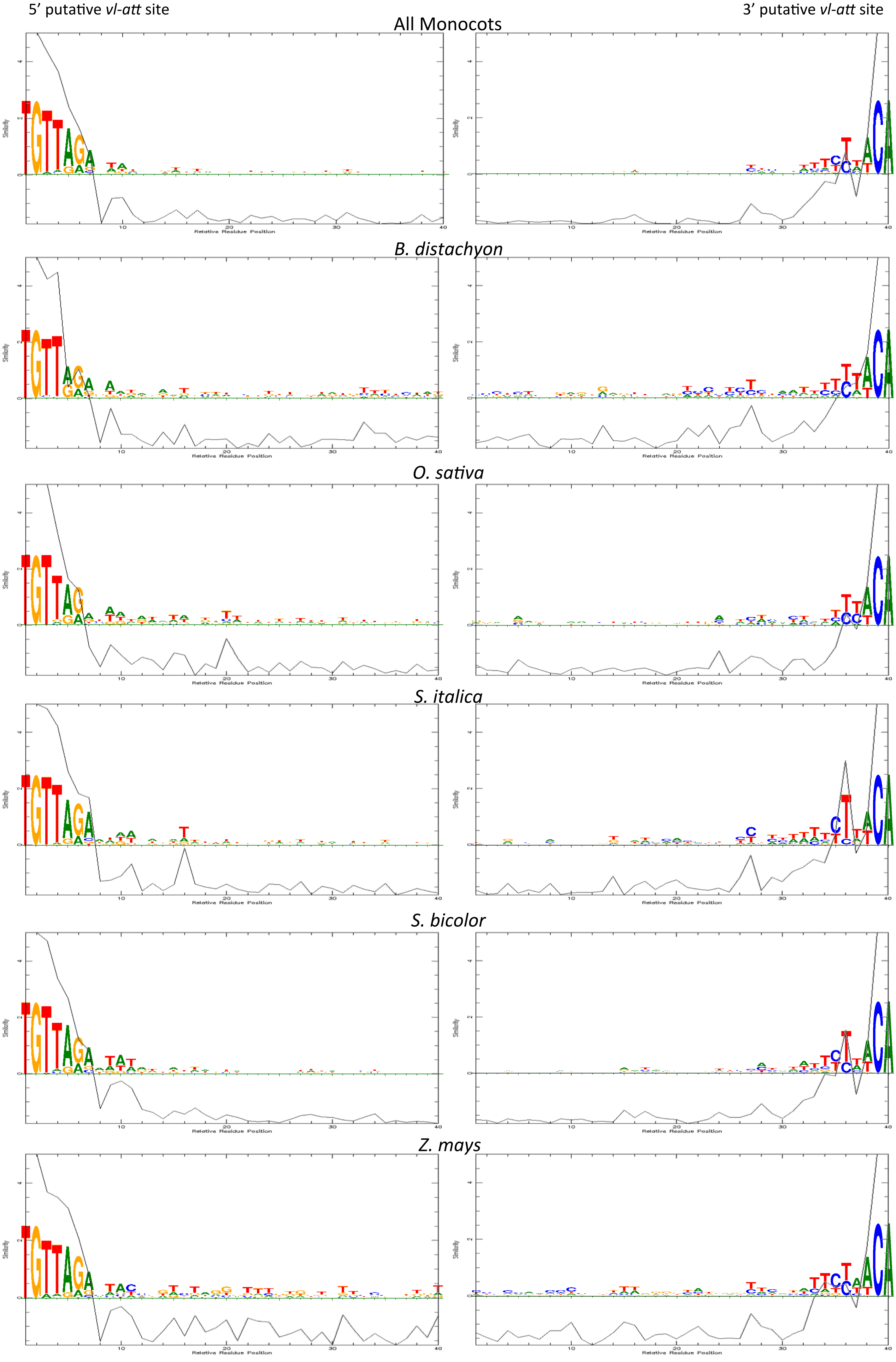

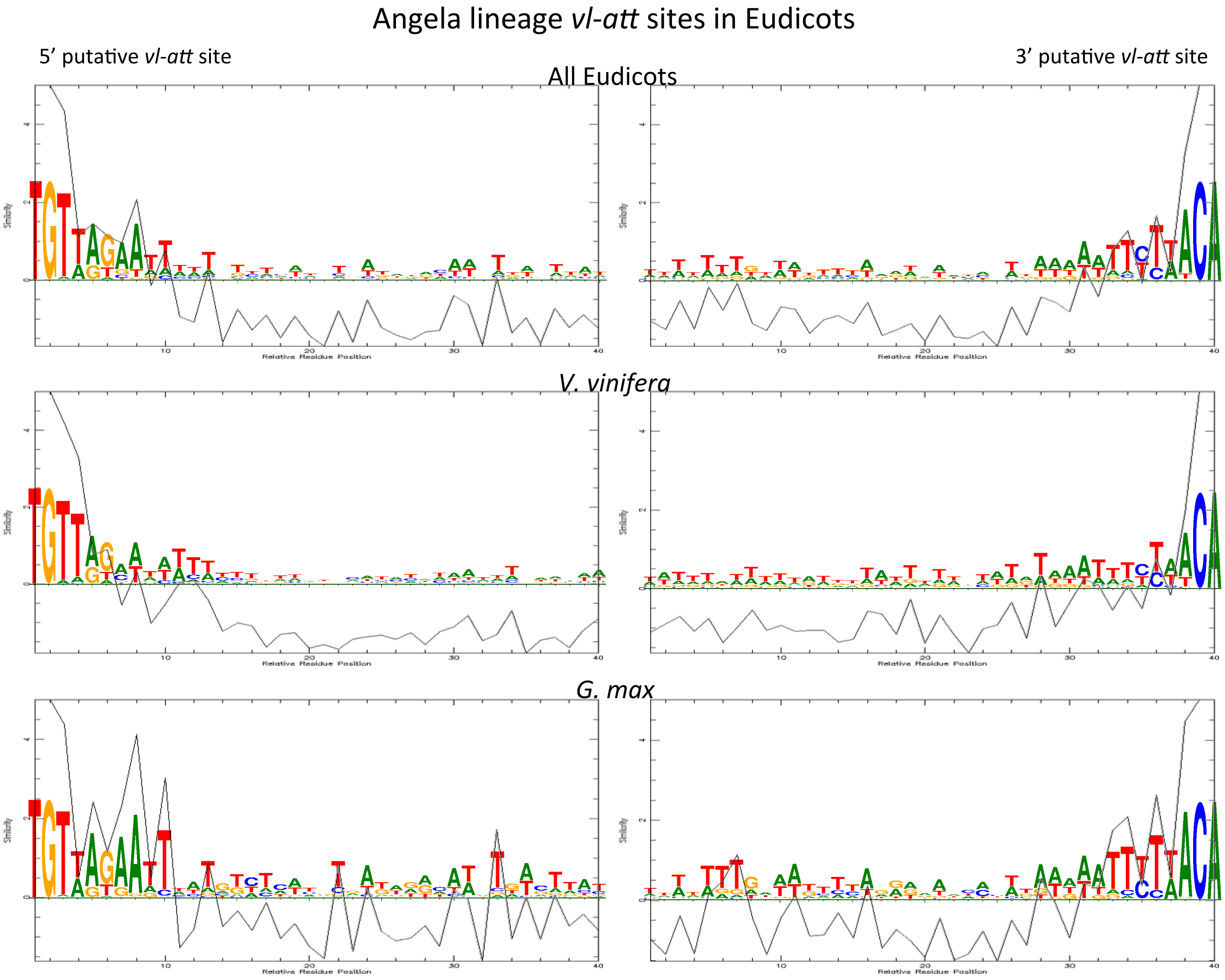
Angela lineage *vl-att* sites in Eudicots
5’ putative *vl-att* site
3’ putative *vl-att* site
All Eudicots
V. vinifera
G. max
Similarity
Relative Residue Position

Angela lineage *vl-att* sites in Monocots

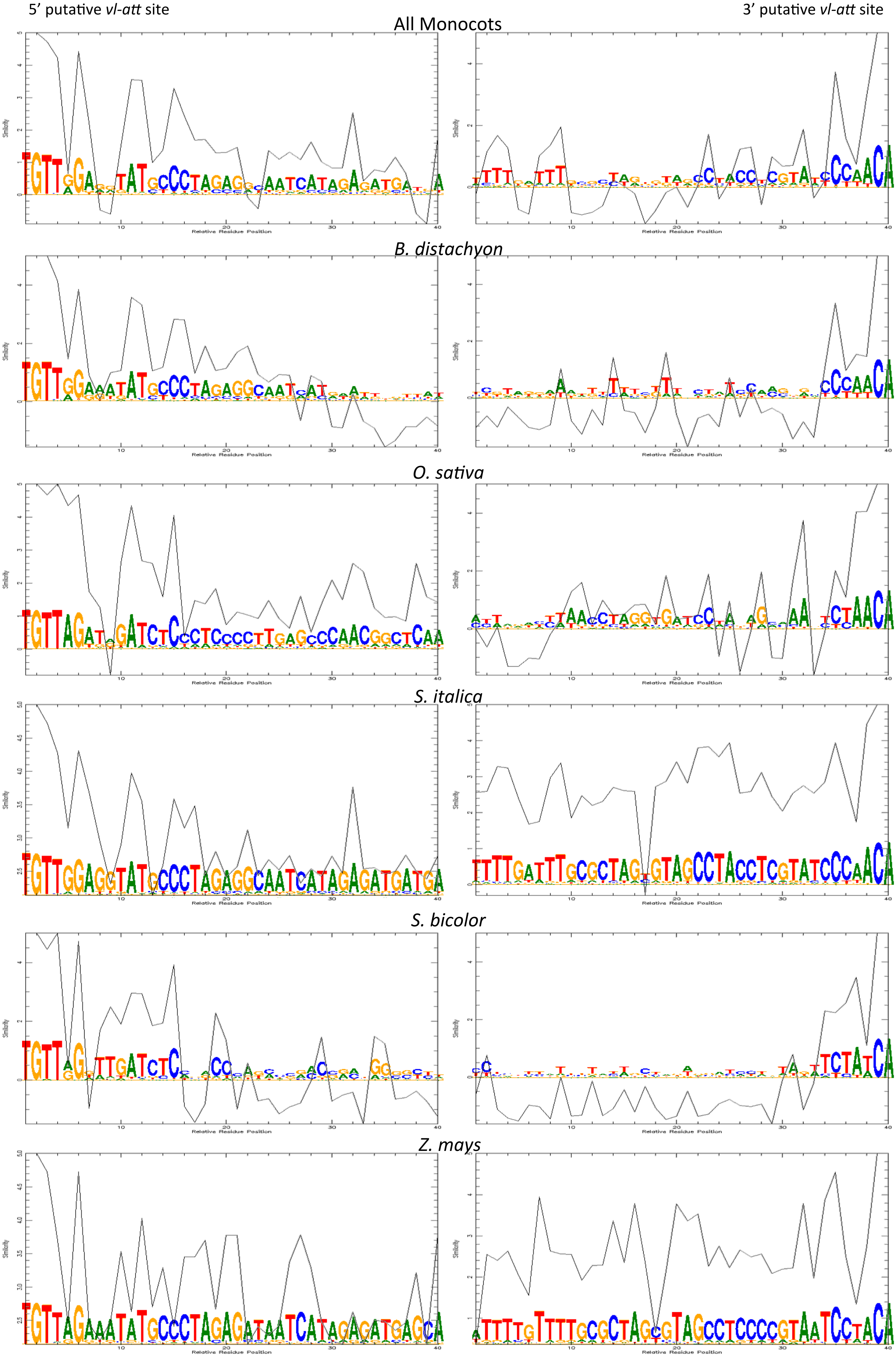

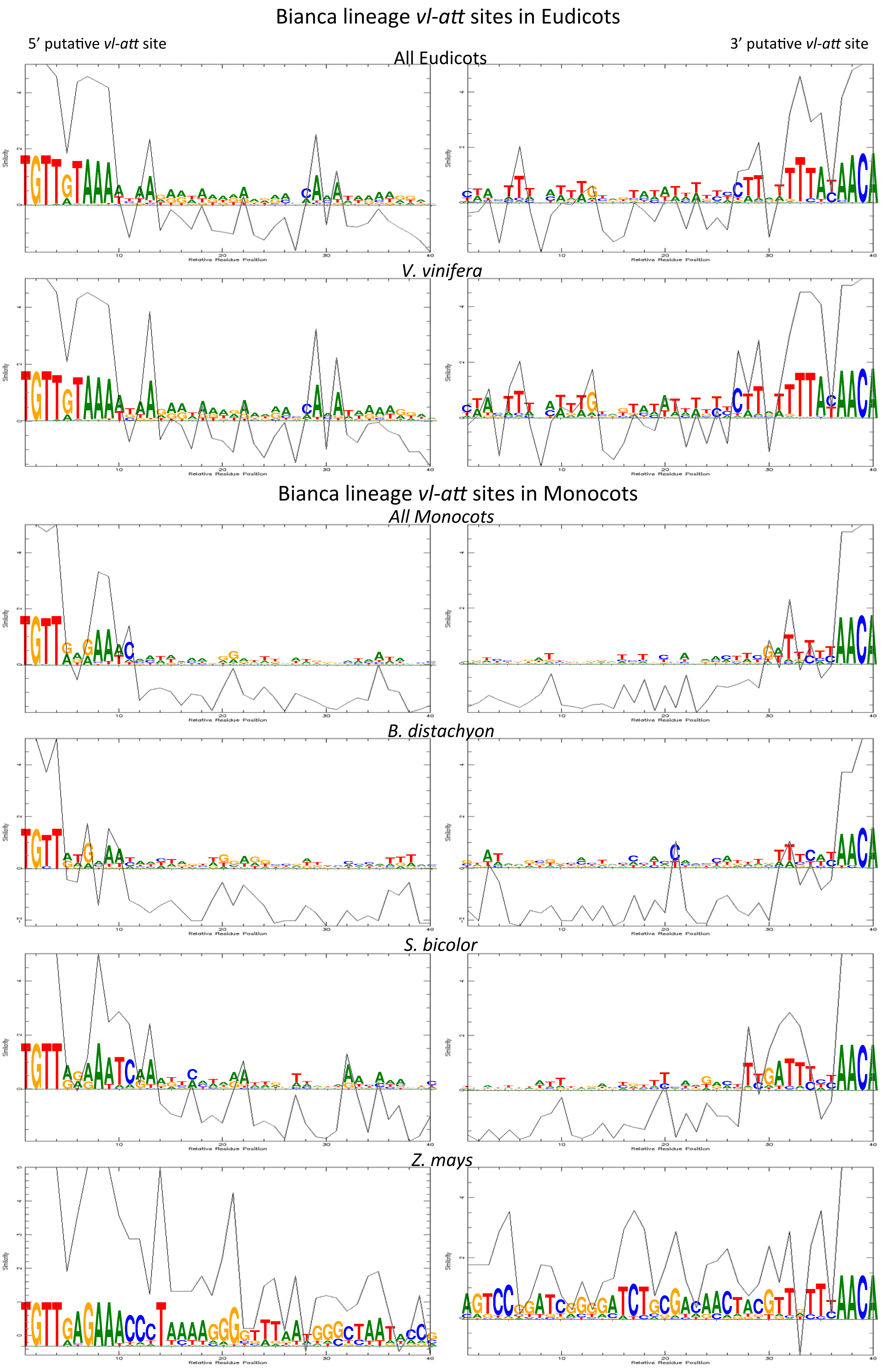
Bianca lineage vl-att sites in Eudicots
5′ putative vl-att site
3′ putative vl-att site
All Eudicots
V. vinifera
Bianca lineage vl-att sites in Monocots
All Monocots
B. distachyon
S. bicolor
Z. mays
Similarity
Relative Residue Position

Ivana lineage *vl-att* sites in Eudicots

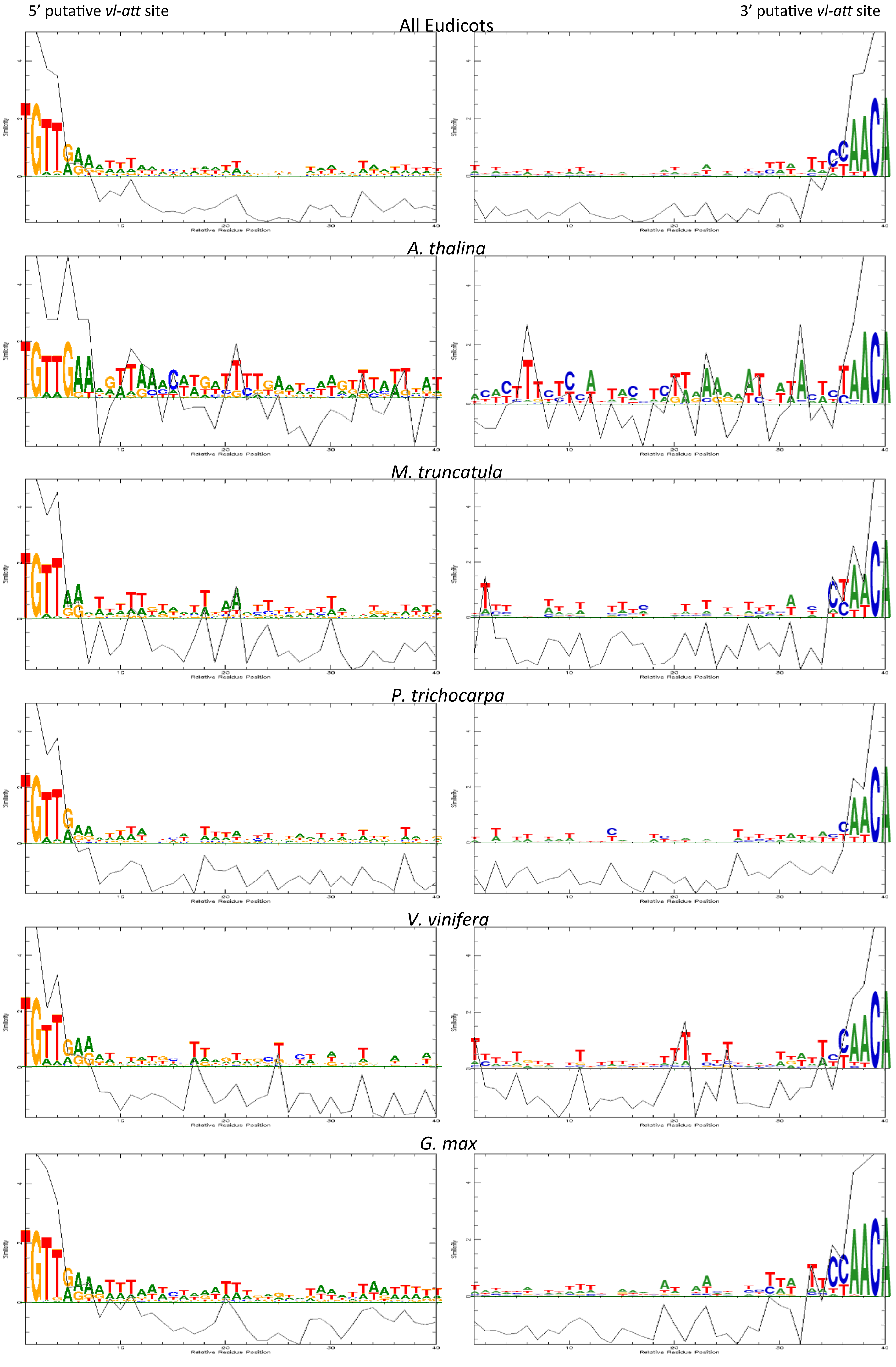

Ivana lineage *vl-att* sites in Monocots

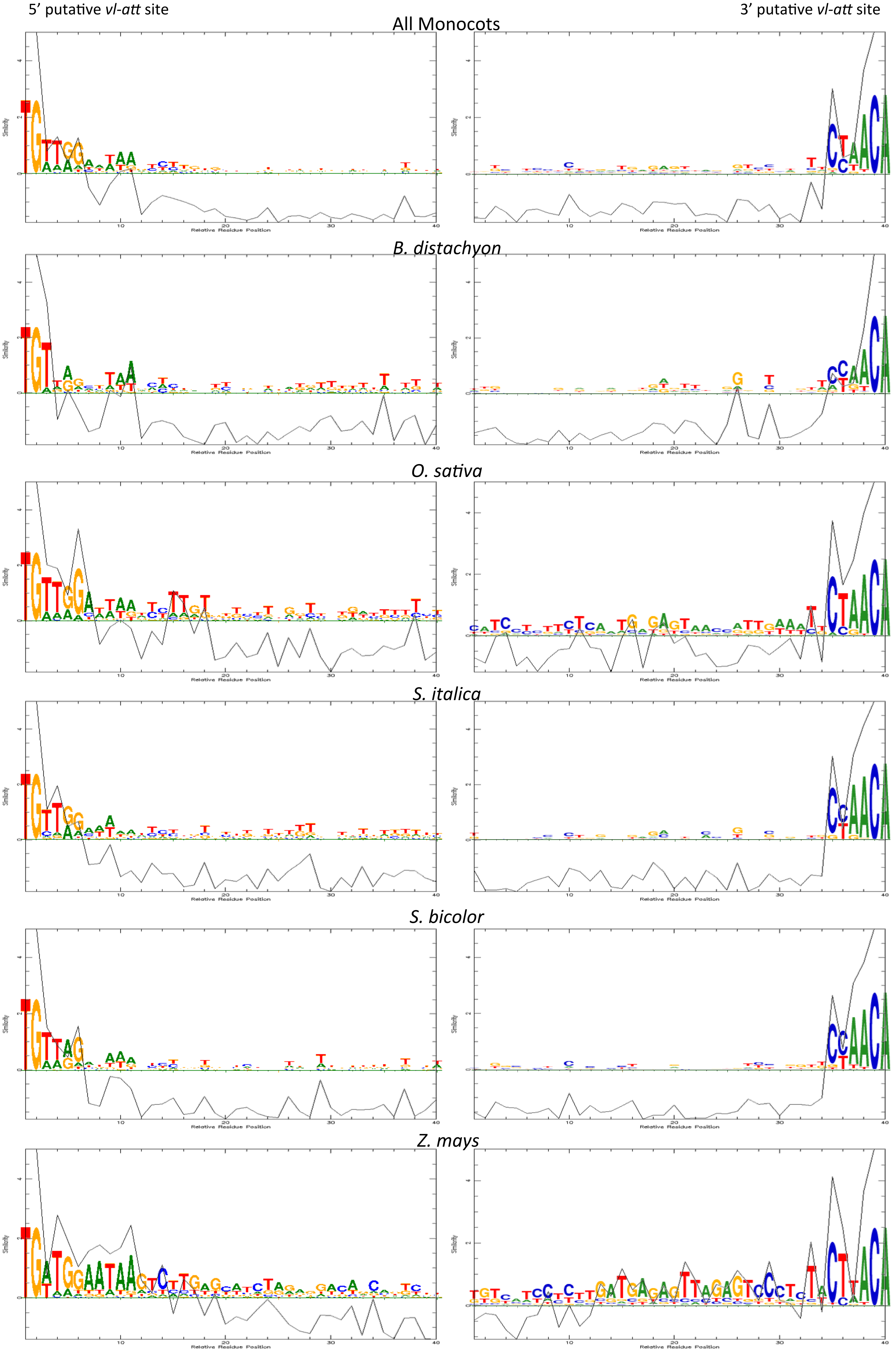

Maximus lineage *vl-att* sites in Eudicots

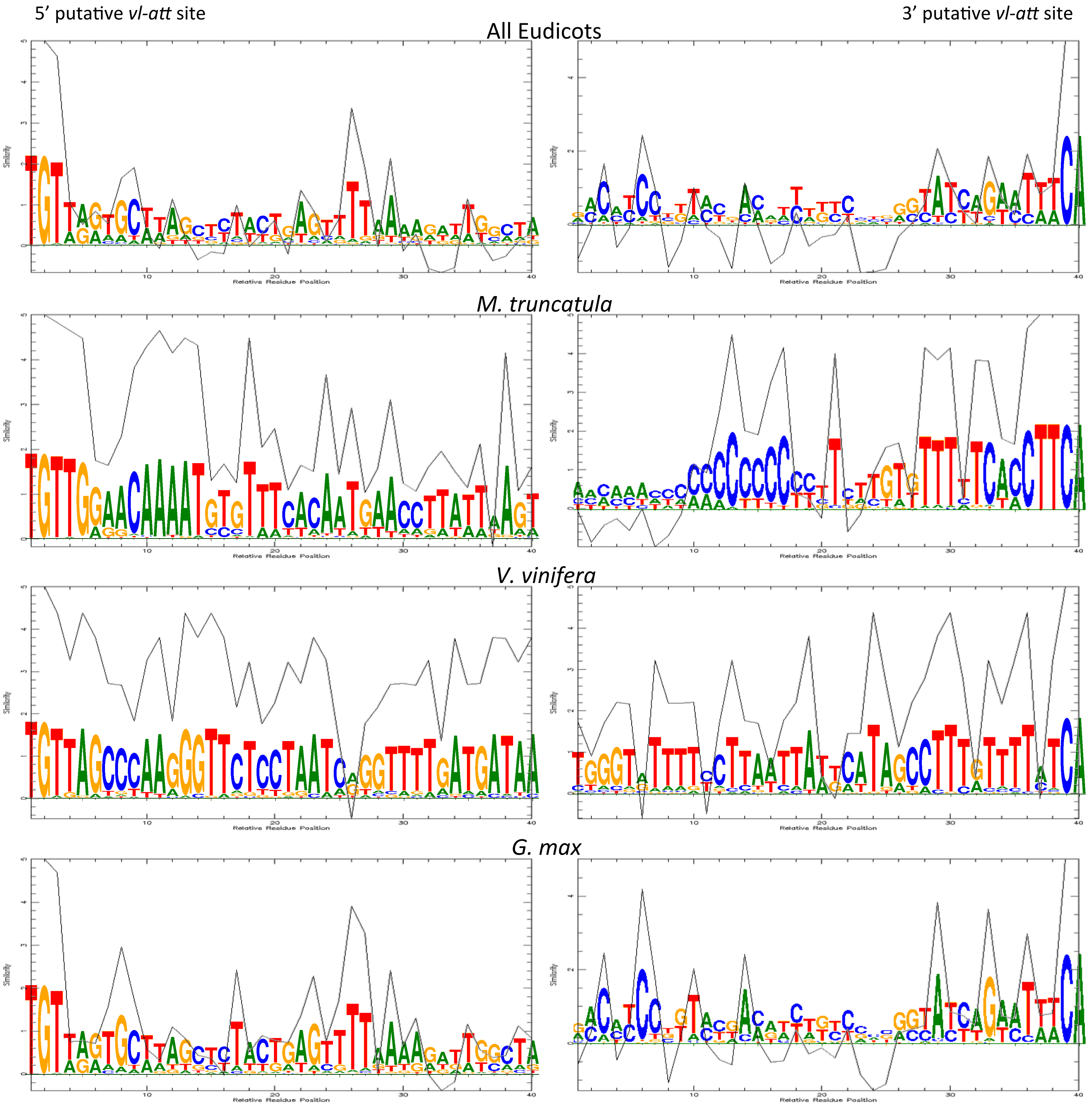

Maximus lineage *vl-att* sites in Monocots

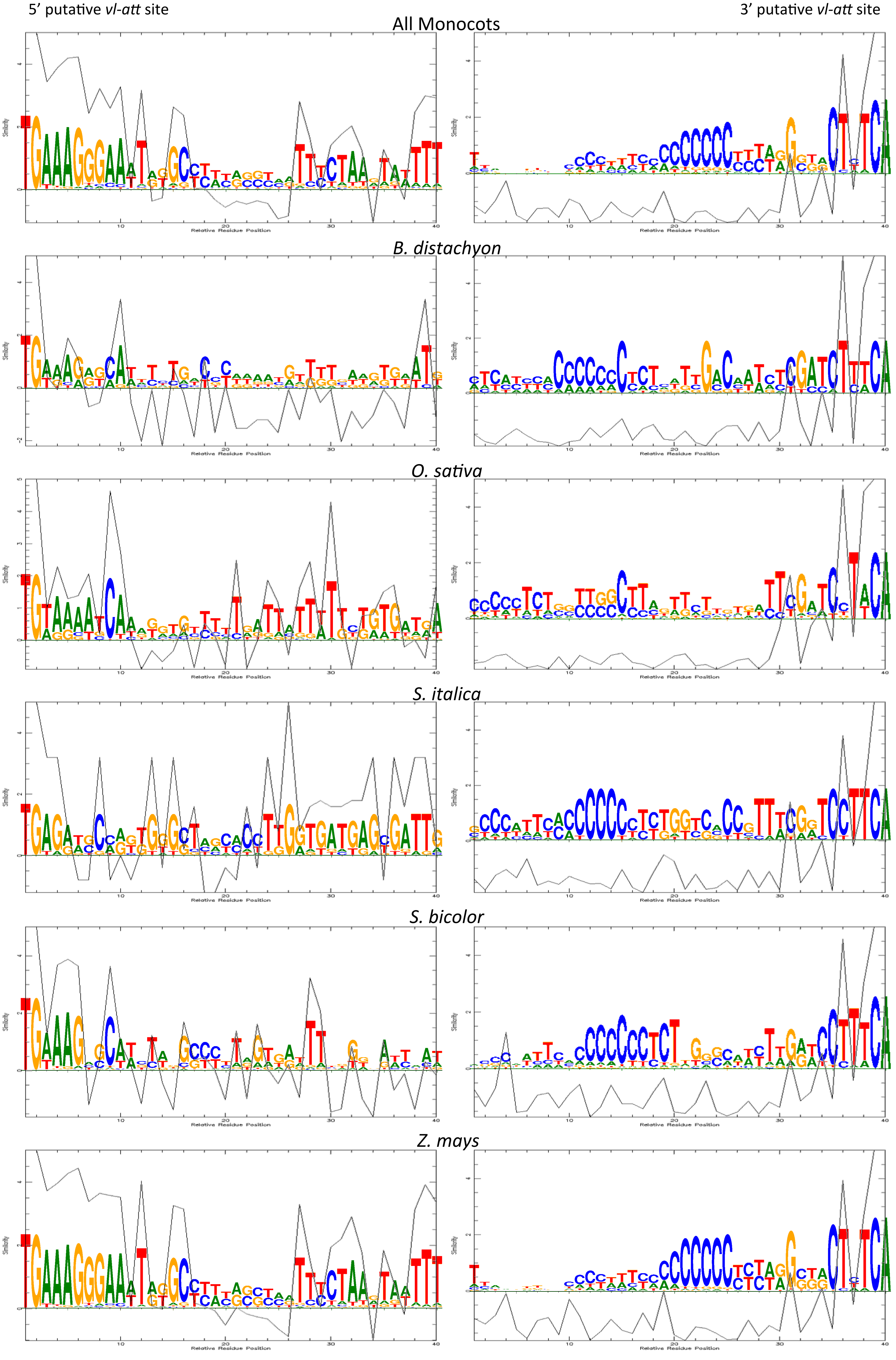

Tar lineage *vl-att* sites in Eudicots

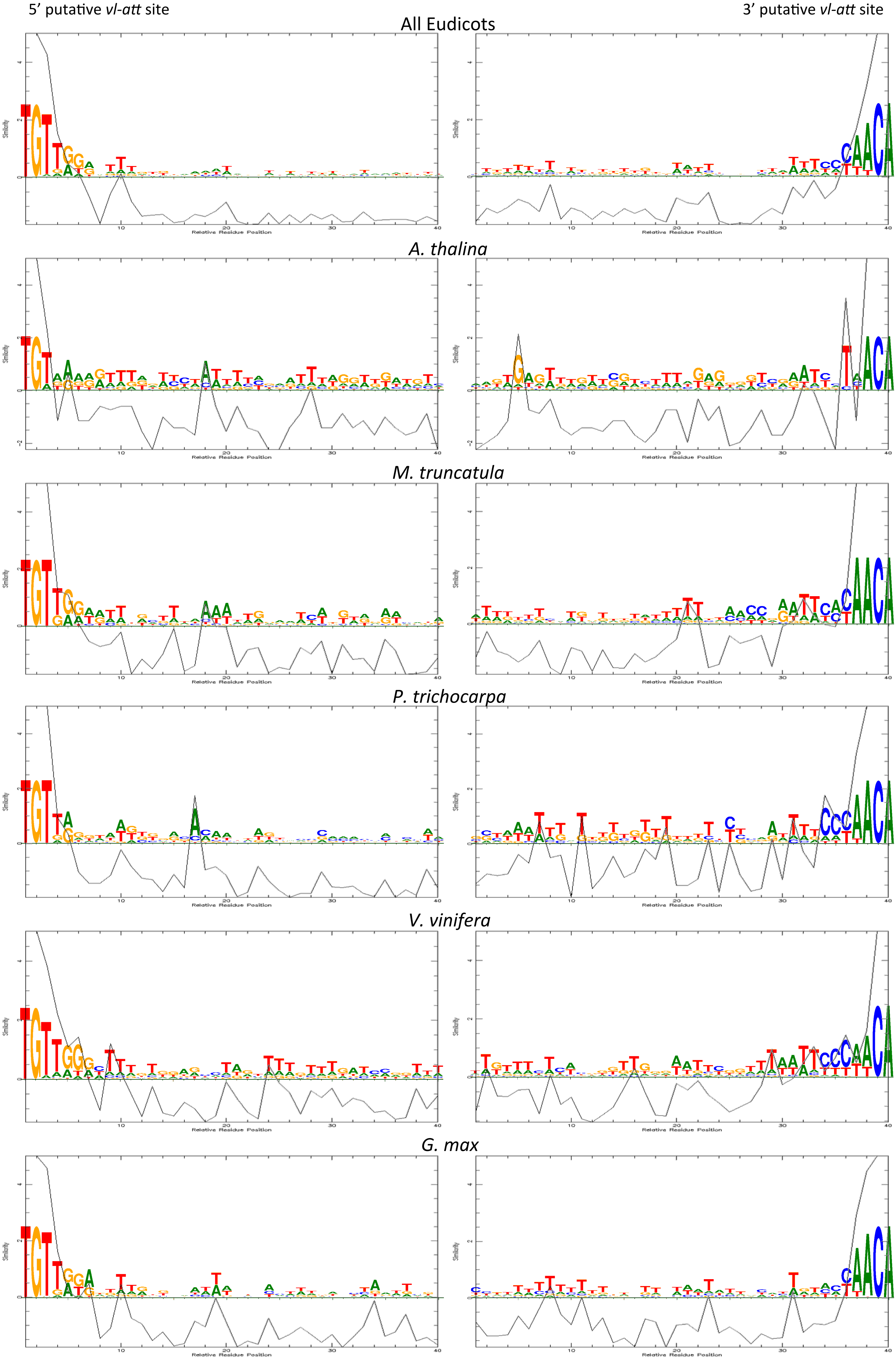

Tar lineage *vl-att* sites in Monocots

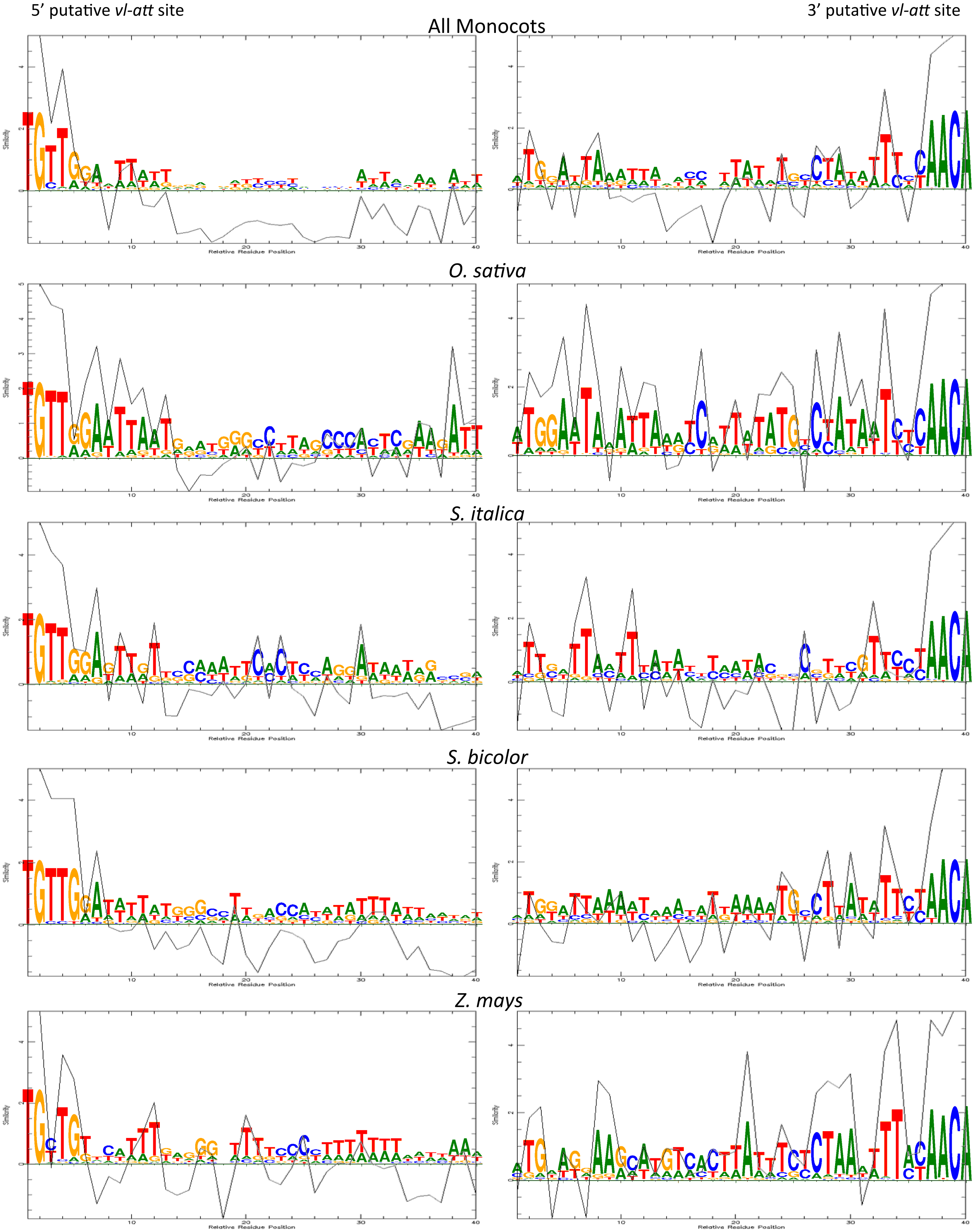

Athila lineage *vl-att* sites in Eudicots

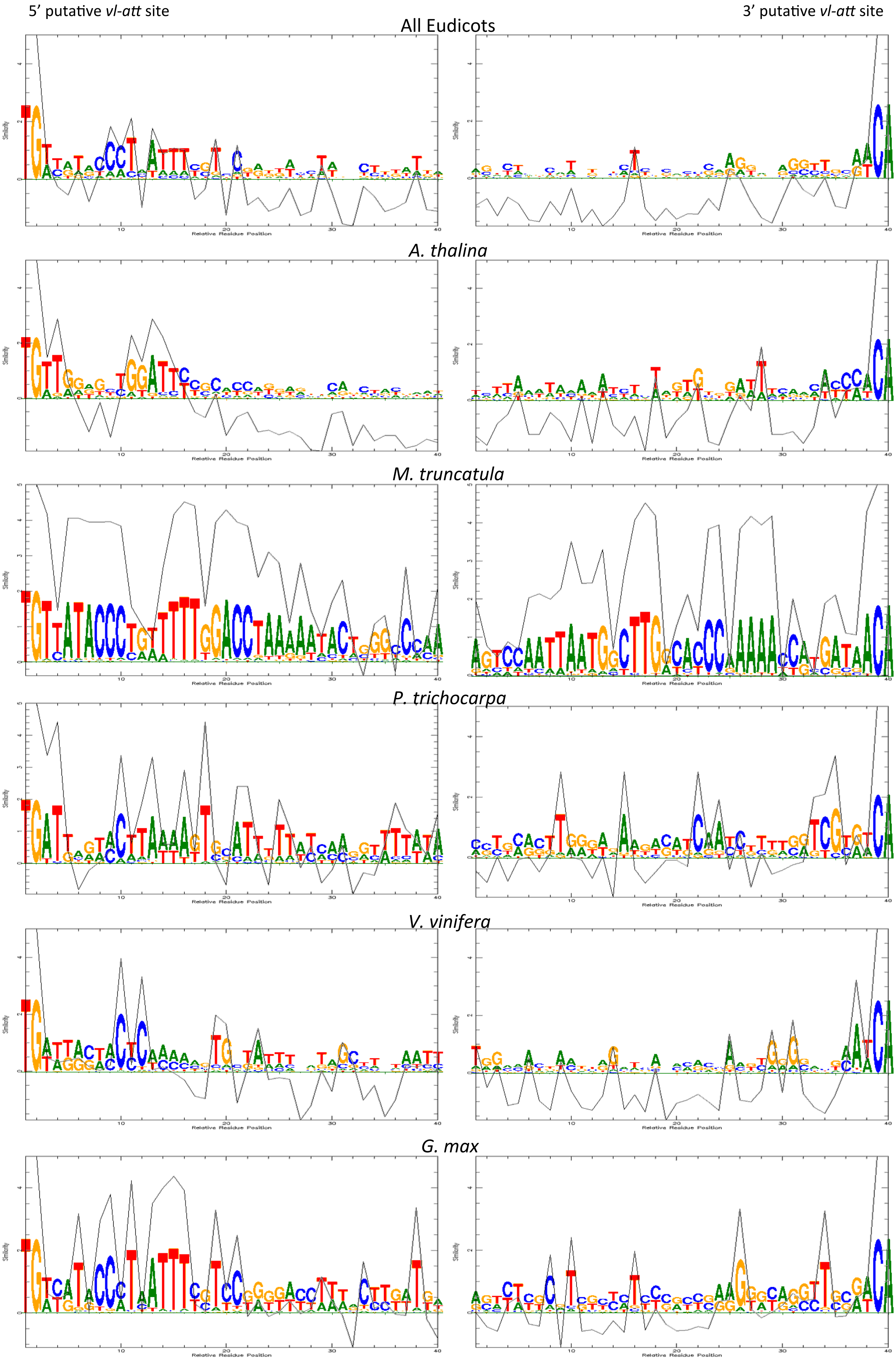

Athila lineage *vl-att* sites in Monocots

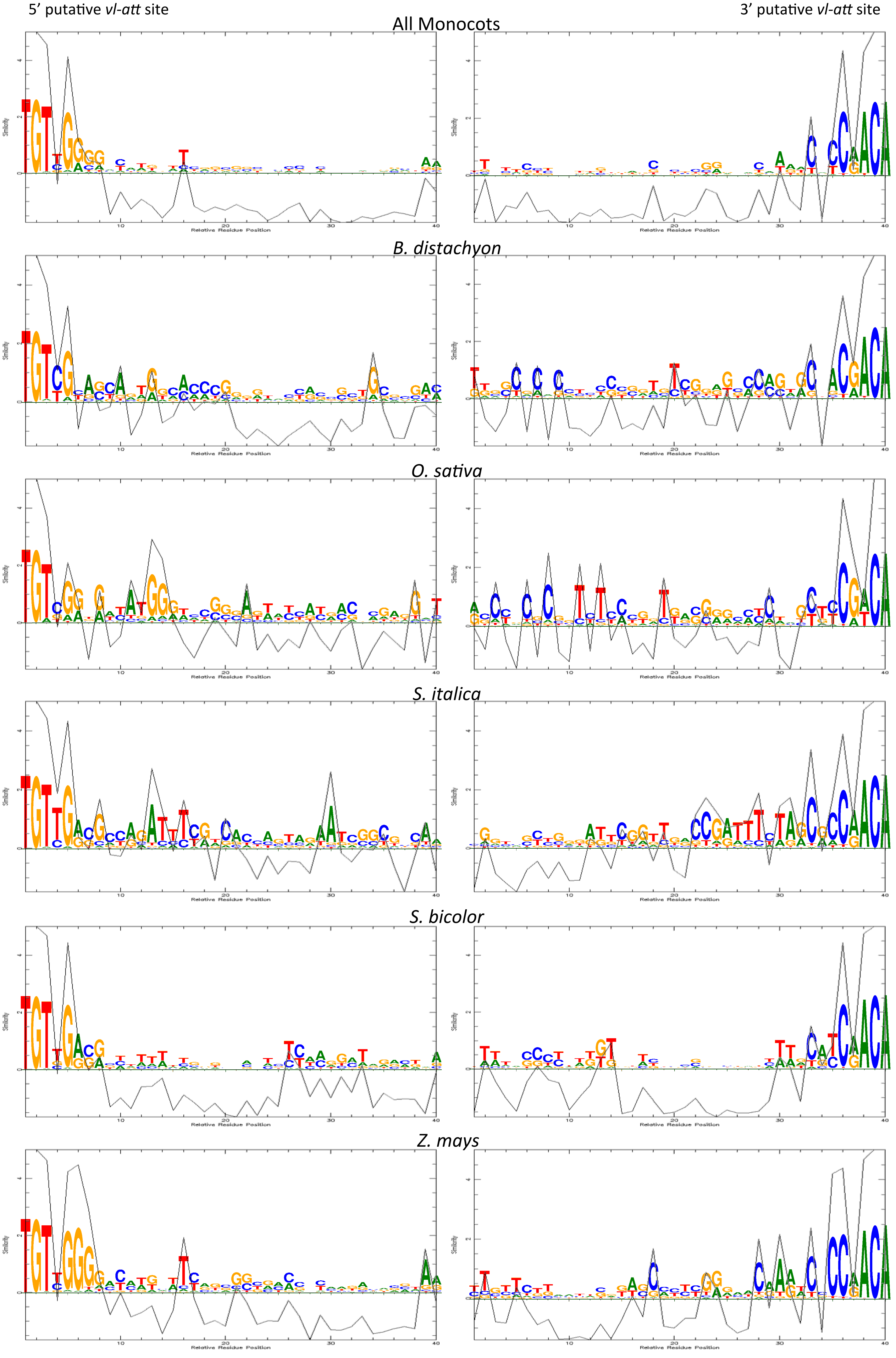

CRM lineage *vl-att* sites in Eudicots

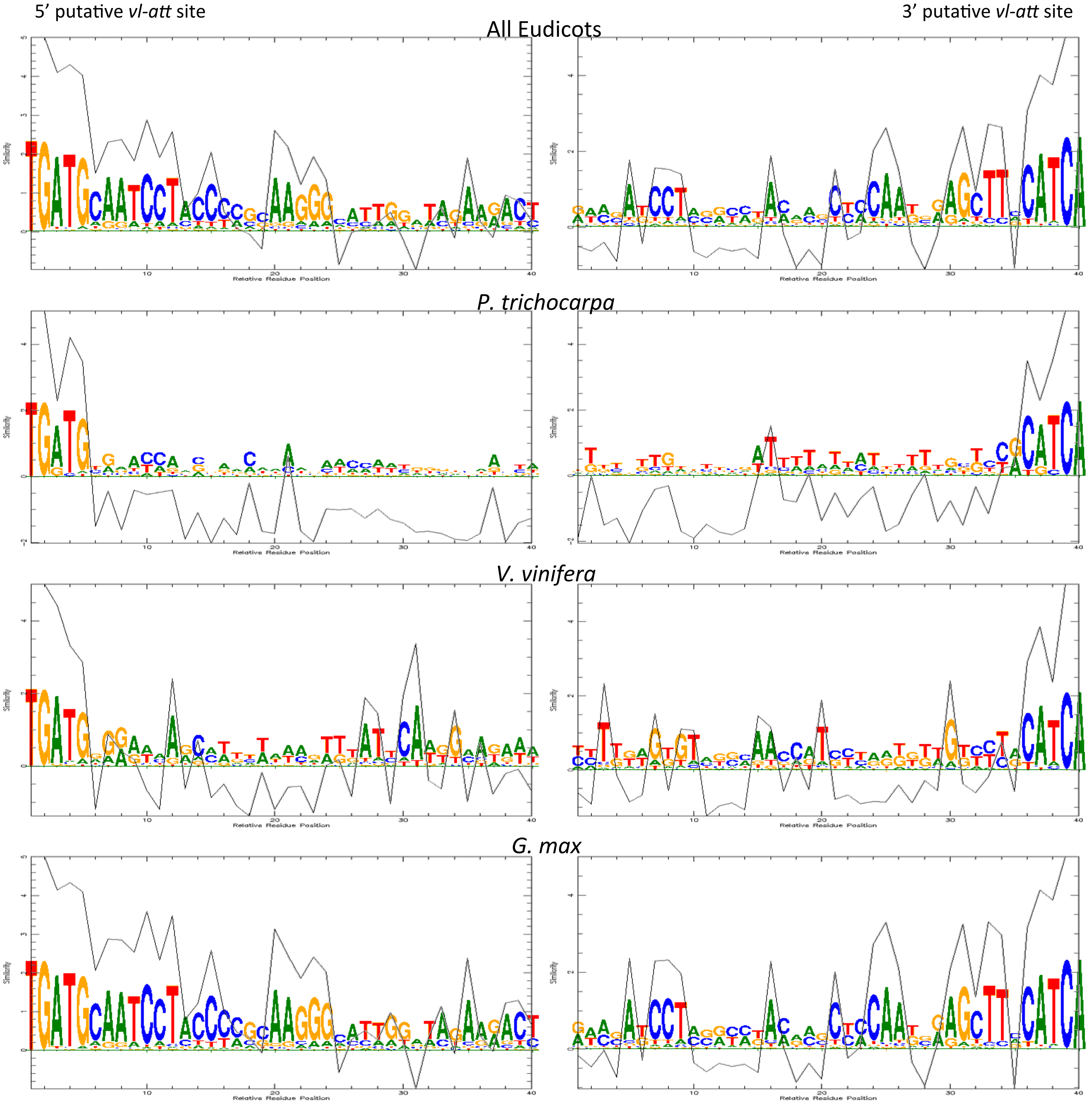

CRM lineage *vl-att* sites in Monocots

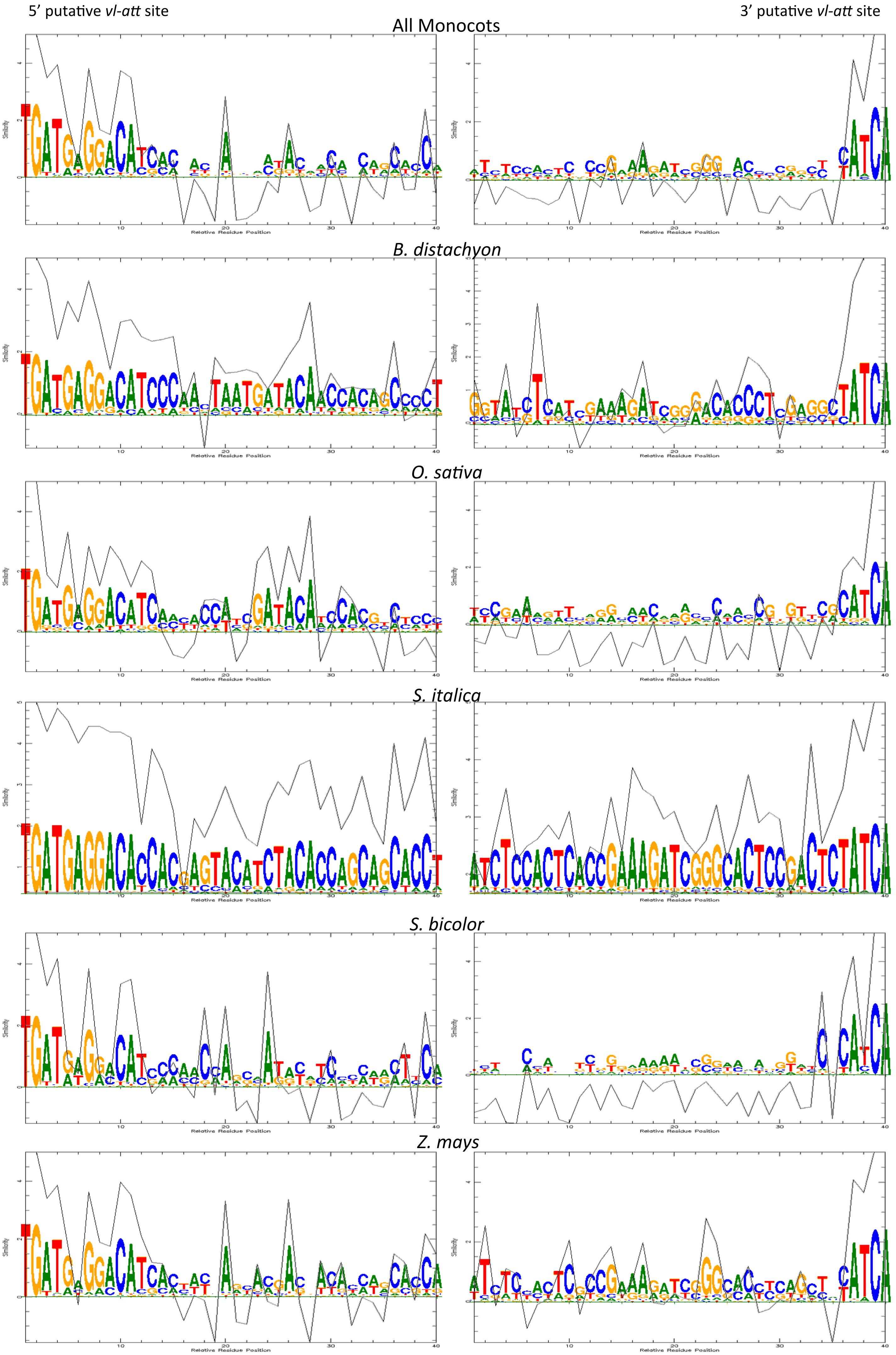

Reina lineage *vl-att* sites in Eudicots

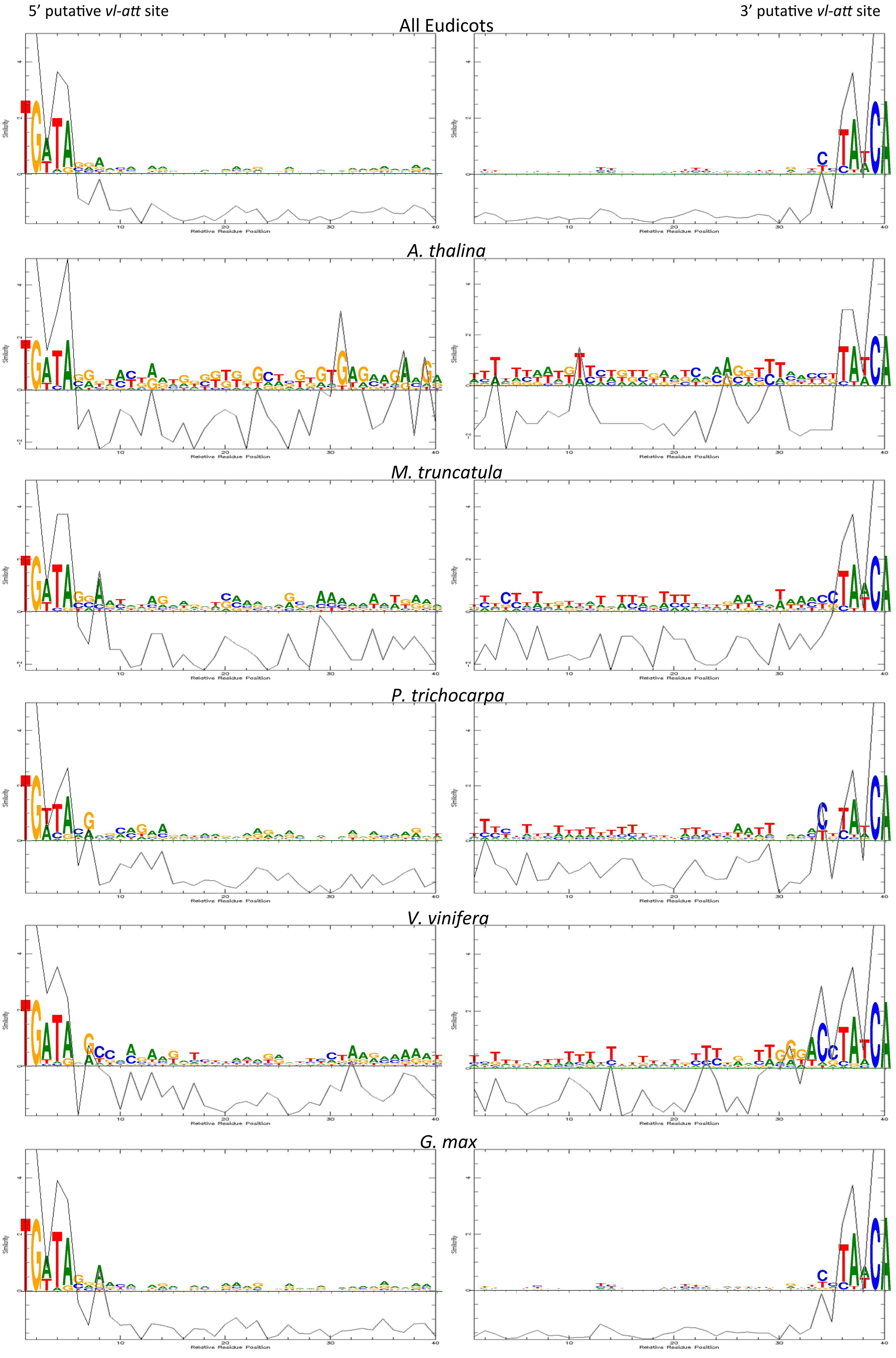

Reina lineage *vl-att* sites in Monocots

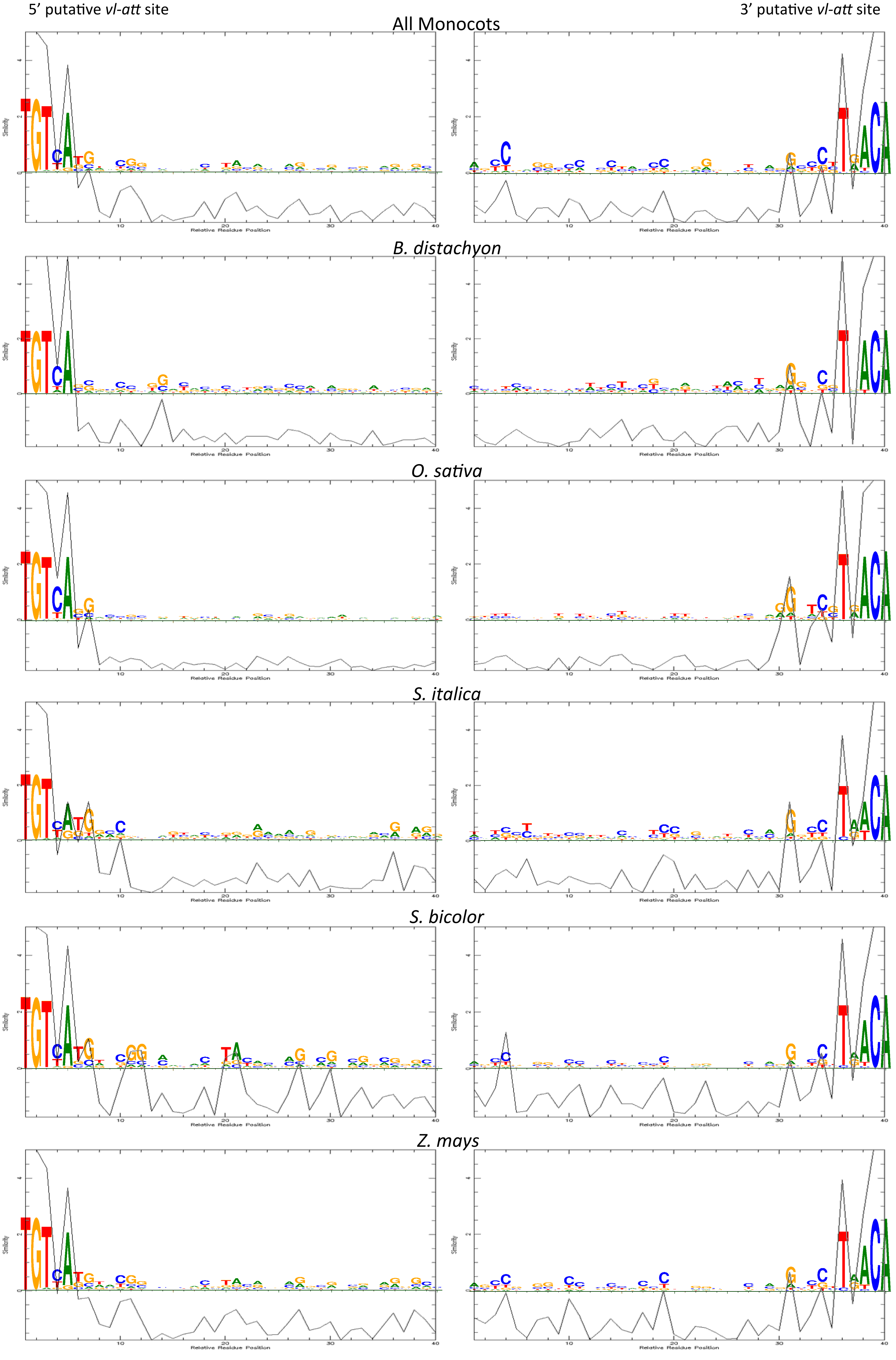

Supplement: Additional file 1: Figure S1. — Sequence logos and PlotCon of U3 and U5 vl-att putative sites of 9 LTR-retrotransposon lineages divided by genome and plant group. Sequence logos of the first and last 40 bases of the LTR from 9 LTR-RT lineages divided by genome or plant group (eudicot - monocot species). Sequence logo is a graphical representation of nucleic acid multiple sequence alignment. Each logo consists of stacks of symbols, one stack for each position in the sequence. The overall height of the stack indicates the sequence conservation at that position, while the height of symbols within the stack indicates the relative frequency of each nucleic acid at that position. Behind each logo it is the PlotCon analysis, where the X-axis for all plots refers to the relative residue position in each alignment and the Y-axis to their similarity, indicated as the pairwise scores that are taken from the specified similarity matrix. The PlotCon graphics are based on an algorithm that shows, along the alignment, the regions with significant similarity (above 0 mark of similarity), giving a strong view of the vl-att sites candidates. (PDF 8527 kb) [file 13100_2016_69_MOESM1_ESM.pdf]
